# Health Risks from Lead-Based Ammunition in the Environment

**DOI:** 10.1289/ehp.1306945

**Published:** 2013-06-01

**Authors:** David C. Bellinger, Joanna Burger, Tom J. Cade, Deborah A. Cory-Slechta, Myra Finkelstein, Howard Hu, Michael Kosnett, Philip J. Landrigan, Bruce Lanphear, Mark A. Pokras, Patrick T. Redig, Bruce A. Rideout, Ellen Silbergeld, Robert Wright, Donald R. Smith

**Affiliations:** 1 Harvard Medical School, Harvard School of Public Health, Boston, Massachusetts; 2 Environmental and Occupational Health Sciences Institute, Rutgers University, Piscataway, New Jersey; 3 Department of Ecology and Evolutionary Biology (Emeritus), Cornell University, Ithaca, New York; 4 Department of Environmental Medicine, University of Rochester School of Medicine, Rochester, New York; 5 Department of Microbiology and Environmental Toxicology, University of California, Santa Cruz, Santa Cruz, California; 6 Dalla Lana School of Public Health, University of Toronto, Toronto, Ontario, Canada; 7 Division of Clinical Pharmacology & Toxicology, Department of Medicine, University of Colorado School of Medicine, Aurora, Colorado; 8 Department of Preventive Medicine, Icahn School of Medicine at Mount Sinai, New York, New York; 9 Child & Family Research Institute, BC Children’s Hospital, Simon Fraser University, Vancouver, British Columbia, Canada; 10 Wildlife Clinic, Department of Infectious Disease and Global Health, Cummings School of Veterinary Medicine, Tufts University, North Grafton, Massachusetts; 11 The Raptor Center, Veterinary Clinical Sciences, University of Minnesota, St. Paul, Minnesota; 12 Wildlife Disease Laboratories, Institute for Conservation Research, San Diego Zoo Global, San Diego, California; 13 Department of Environmental Health Sciences, Johns Hopkins Bloomberg School of Public Health, Baltimore, Maryland; 14 Department of Preventive Medicine, Icahn School of Medicine at Mount Sinai, New York, New York; 15 Department of Microbiology and Environmental Toxicology, University of California, Santa Cruz, Santa Cruz, California, E-mail: drsmith@ucsc.edu

Lead is one of the most studied toxicants, and overwhelming scientific evidence demonstrates that lead is toxic to several physiological systems in vertebrates, including the nervous, renal, cardiovascular, reproductive, immune, and hematologic systems (Health Risks from Lead-Based Ammunition in the Environment—A Consensus Statement of Scientists 2013). Furthermore, there is no level of lead exposure in children known to be without adverse effects [Centers for Disease Control and Prevention (CDC) 2012a, 2012b].

In light of this evidence, there is an urgent need to end a major source of lead for animals and humans: spent lead bullets and shotgun pellets. Notably, production of lead-based ammunition in the United States accounted for > 69,000 metric tons consumed in 2012; this is second only to the amount of lead used to manufacture storage batteries (U.S. Geological Survey 2013). However, there are few regulations regarding the release of lead into the environment through discharge of lead-based ammunition. For other major categories of lead consumption, such as lead batteries and sheet lead/lead pipes, environmental discharge and disposal are regulated. Therefore, lead-based ammunition is likely the greatest largely unregulated source of lead that is knowingly discharged into the environment in the United States. In contrast, the release or distribution of other major sources of environmental lead contamination (e.g., leaded gasoline, lead-based paint, lead solder) have been substantially regulated and reduced since the mid-1970s (Health Risks from Lead-Based Ammunition in the Environment—A Consensus Statement of Scientists 2013).

There is a national discussion—polarized at times—of the health risks posed to humans and wildlife from the discharge of lead-based ammunition. To inform this discussion, a group of 30 nationally and internationally recognized scientists with expertise regarding lead and environmental health recently collaborated to create an evidence-based consensus statement (Health Risks from Lead-Based Ammunition in the Environment—A Consensus Statement of Scientists 2013) supporting the reduction and eventual elimination of lead released to the environment through the discharge of lead-based ammunition.

The discharge of lead bullets and shotgun pellets into the environment poses significant health risks to humans and wildlife. The best available scientific evidence demonstrates that the discharge of lead-based ammunition substantially increases environmental lead levels, especially in areas with higher shooting activity (U.S. Environmetal Protection Agency 2012) and that the discharge of lead-based ammunition poses risks of elevated lead exposure to gun users (National Research Council 2012). When lead-containing bullets are used to shoot wildlife, they can fragment into hundreds of small pieces, many of which are small enough to be easily ingested by scavenging animals or to be retained in meat prepared for human consumption (Hunt et al. 2009; Knott et al. 2010; Pauli and Burkirk 2007). Consequently, lead-based ammunition may be a significant source of lead exposure in humans that regularly ingest wild game (Hanning et al. 2003; Johansen et al. 2006; Levesque et al. 2003; Tsuji et al. 2008). In addition, lead pellets and fragments have been reported in gastrointestinal tracts of hunters who consume meat from animals shot with lead-based ammunition (Carey 1977; Reddy 1985).

The use of lead pellets in shotgun shells for hunting waterfowl posed a serious threat to wetland birds, and secondarily to bald eagles, in the United States, leading to the U.S. Fish and Wildlife Service’s 1991 nationwide regulations requiring use of nontoxic shotgun pellets for hunting waterfowl (Anderson 1992). However, lead poisoning from ingestion of spent lead-based ammunition fragments continues to pose a particularly serious health threat for scavenging species. These lead-containing fragments remain the principal source of lead exposure to endangered California condors and continue to prevent the successful recovery of these birds in the wild (Church et al. 2006; Finkelstein et al. 2012; Green et al. 2008; Parish et al. 2009; Rideout et al. 2012; Woods et al. 2007). Other wildlife species, such as golden eagles, bald eagles, ravens, turkey vultures, and pumas, are also exposed to the fragments of spent lead ammunition (Burco et al. 2012; Clark and Scheuhammer 2003; Craighead and Bedrosian 2008; Cruz-Martinez et al. 2012; Fisher et al. 2006; Kelly and Johnson 2011; Stauber et al. 2010; Wayland and Bollinger 1999).

No rational deliberation about the use of lead-based ammunition can ignore the overwhelming evidence for the toxic effects of lead, or that the discharge of lead bullets and shot into the environment poses significant risks of lead exposure to humans and wildlife. Given the availability of non-lead ammunition for shooting and hunting (Thomas 2013), the use of lead-based ammunition that introduces lead into the environment can be reduced and eventually eliminated. This seems to be a reasonable and equitable action to protect the health of humans and wildlife.
